# Supplementary material for: Change of surfactant protein D and A after renal ischemia reperfusion injury
Source: PLoS One. 2019 Dec 26;14(12):e0227097. doi: 10.1371/journal.pone.0227097 (PMC6932791; doi:10.1371/journal.pone.0227097)
Supplement: S1 Appendix — (DOCX) [file pone.0227097.s001.docx]

**S1 Appendix. Supplementary materials and methods**

**Surgical protocol**

Briefly, under anesthesia, the kidneys were exposed by a midline incision, and the bilateral renal pedicles were occluded for 23 min using nontraumatic microaneurysm clamps, and core body temperature was maintained using a homeothermic pad. After clamp removal, the abdomen was closed. Sham-operated mice received an identical surgical procedure, except for the occlusion of renal pedicles.

**Collection and preparation of serum samples**

To ensure uniformity, all samples were processed identically. Before the first centrifugation at 3000×g for 10 min, blood was allowed to clot at room temperature for 2 hours. Serum was collected from the supernatant of the centrifuged blood and again centrifuged at 3000×g for 1 min to eliminate red blood cells and finally, stored at -70°C for further use. Samples with notable hemolysis were discarded.

**Preparation of lung lysate samples for ELISA and immunoblot**

Frozen lung was homogenized by TissueRuptor (QIAGEN) on ice in 500 μl of phosphate buffer solution that contained 1% protease inhibitor cocktail (Thermo Fisher). A total of 125 μl RIPA (Radio-Immunoprecipitation Assay) Buffer (Sigma Aldrich) was added, and samples were vortexed, frozen and thawed, and incubated on ice for 20 min. Finally, samples were centrifuged at 4°C at 14,000×g for 15 min; the supernatant was collected and stored at -70°C for downstream process.

**Immunoblotting analyses of lung tissue**

30 µg protein were separated by 10% SDS-PAGE and were then transferred to nitrocellulose membranes. The membranes with proteins were blocked with 5% dry skim milk for 2 and next it was incubated overnight with the primary antibodies at 4°C. The bands on the membranes were visualized using chemiluminescence with Pierce ECL Western Blotting Substrate reagents (Thermo Fisher Scientific, Inc.).
