# Supplementary figures and images for: Change of surfactant protein D and A after renal ischemia reperfusion injury
Source: PLoS One. 2019 Dec 26;14(12):e0227097. doi: 10.1371/journal.pone.0227097 (PMC6932791; doi:10.1371/journal.pone.0227097)

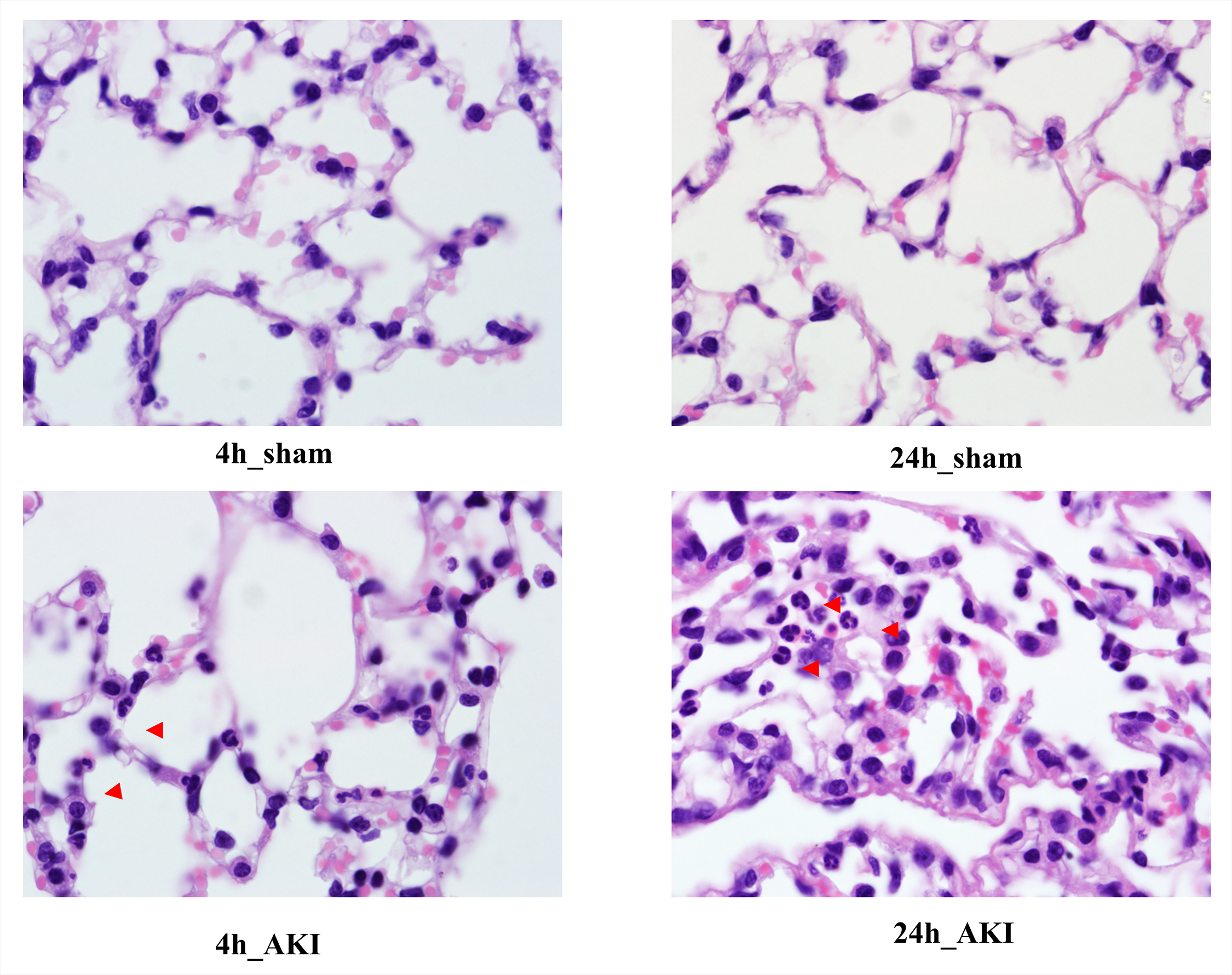

Supplement: S1 Fig — (TIF) [file pone.0227097.s002.tif]
