# Supplementary material for: Change of surfactant protein D and A after renal ischemia reperfusion injury
Source: PLoS One. 2019 Dec 26;14(12):e0227097. doi: 10.1371/journal.pone.0227097 (PMC6932791; doi:10.1371/journal.pone.0227097)
Supplement: S1 Raw Images — (PDF) [file pone.0227097.s003.pdf]

Title: Change of surfactant protein D and A after renal ischemia reperfusion injury

Authors: Islam Md Imtiazul[1], Redwan Asma[1], Ji-Hye Lee[2], Nam-Jun Cho[3], Samel Park[3], Ho-yeon Song[1], Hyo-Wook Gil[3*]

[1]Department of Microbiology, College of Medicine, Soonchunhyang University, Cheonan, Republic of Korea

[2]Department of Pathology, Soonchunhyang University Cheonan Hospital, Cheonan, Republic of Korea

[3]Department of Internal Medicine, Soonchunhyang University Cheonan Hospital, Cheonan, Republic of Korea

* Corresponding author

E-mail: hwgil@schmc.ac.kr

Original Western Blot Images

Figure 3B: BAX

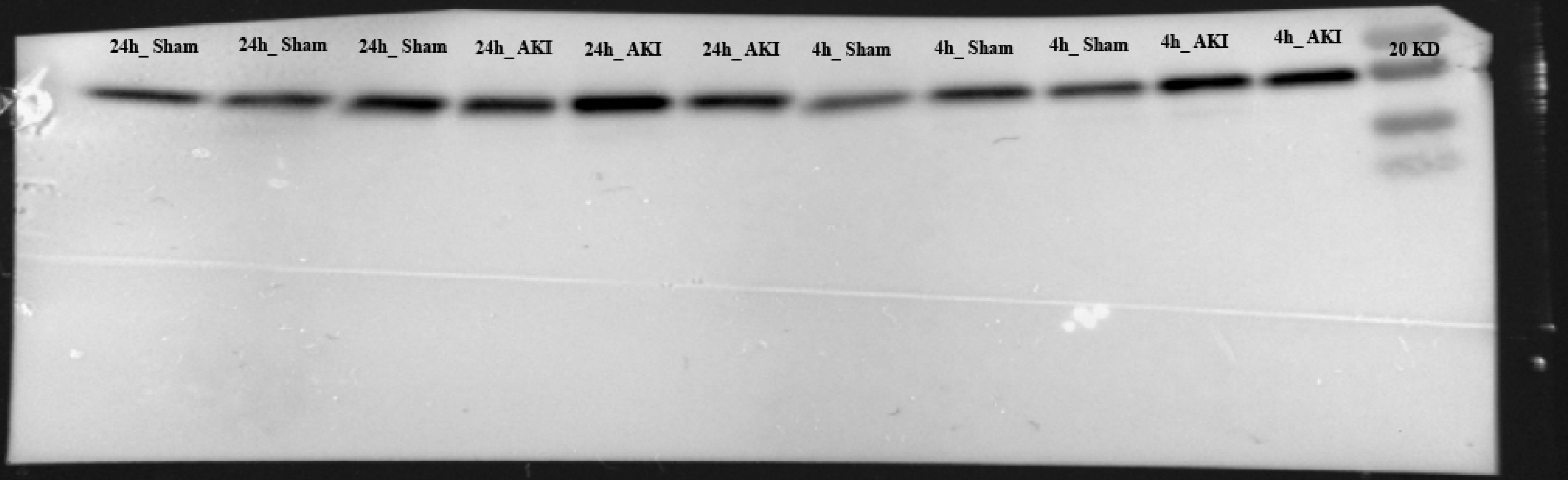

Figure 3B: Bcl-2

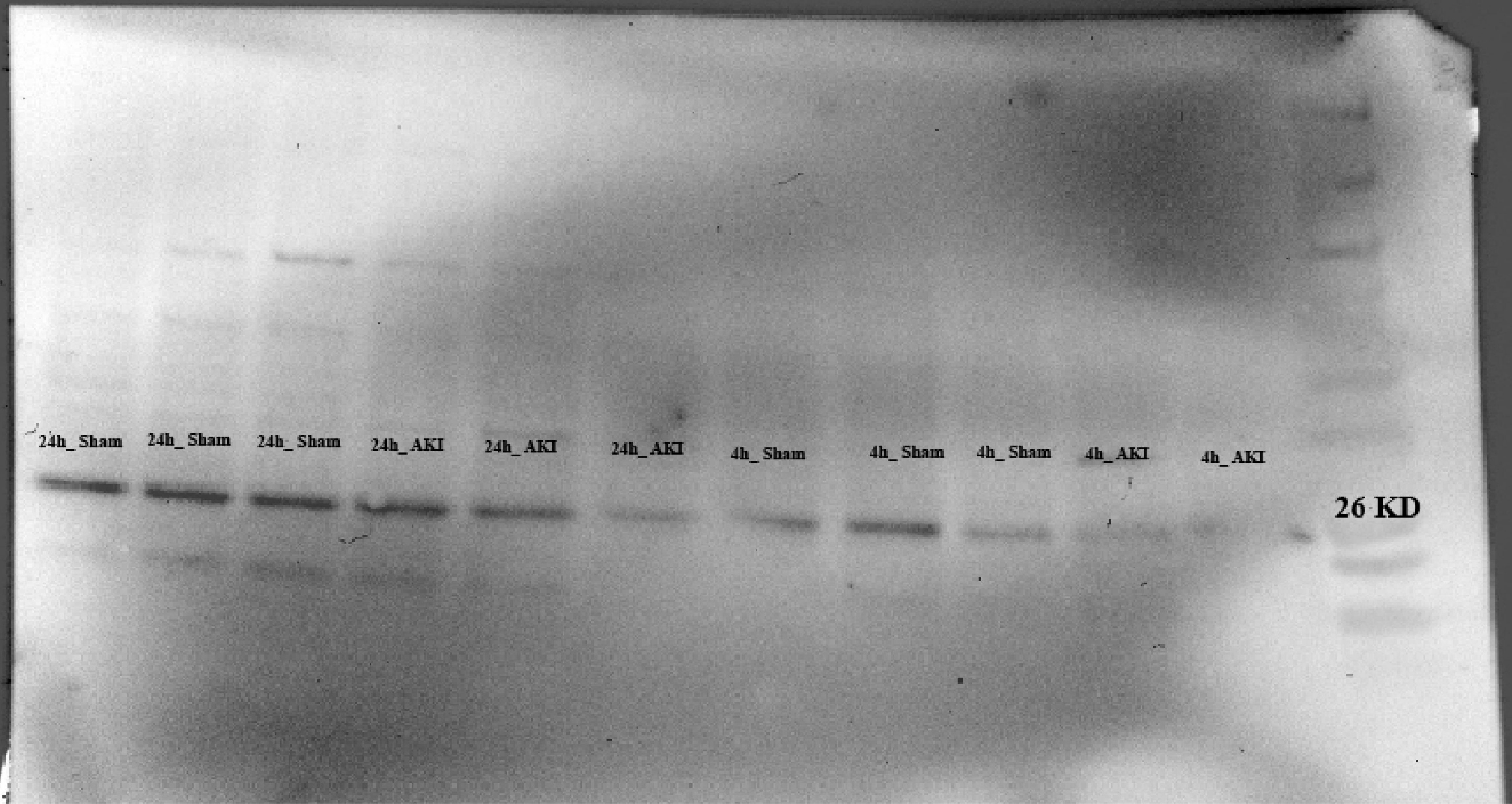

Figure 3B: Beta actin

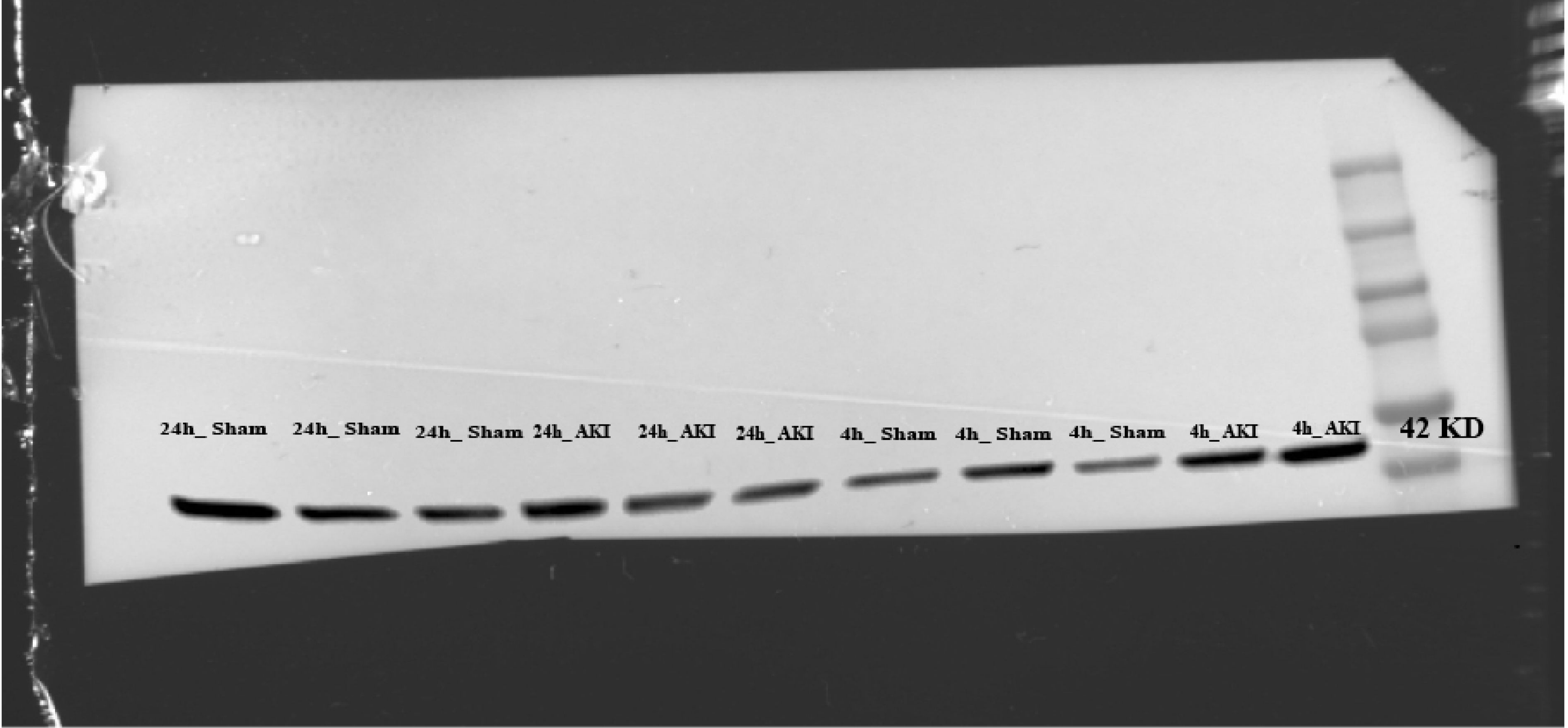

Figure 4: Claudin-3

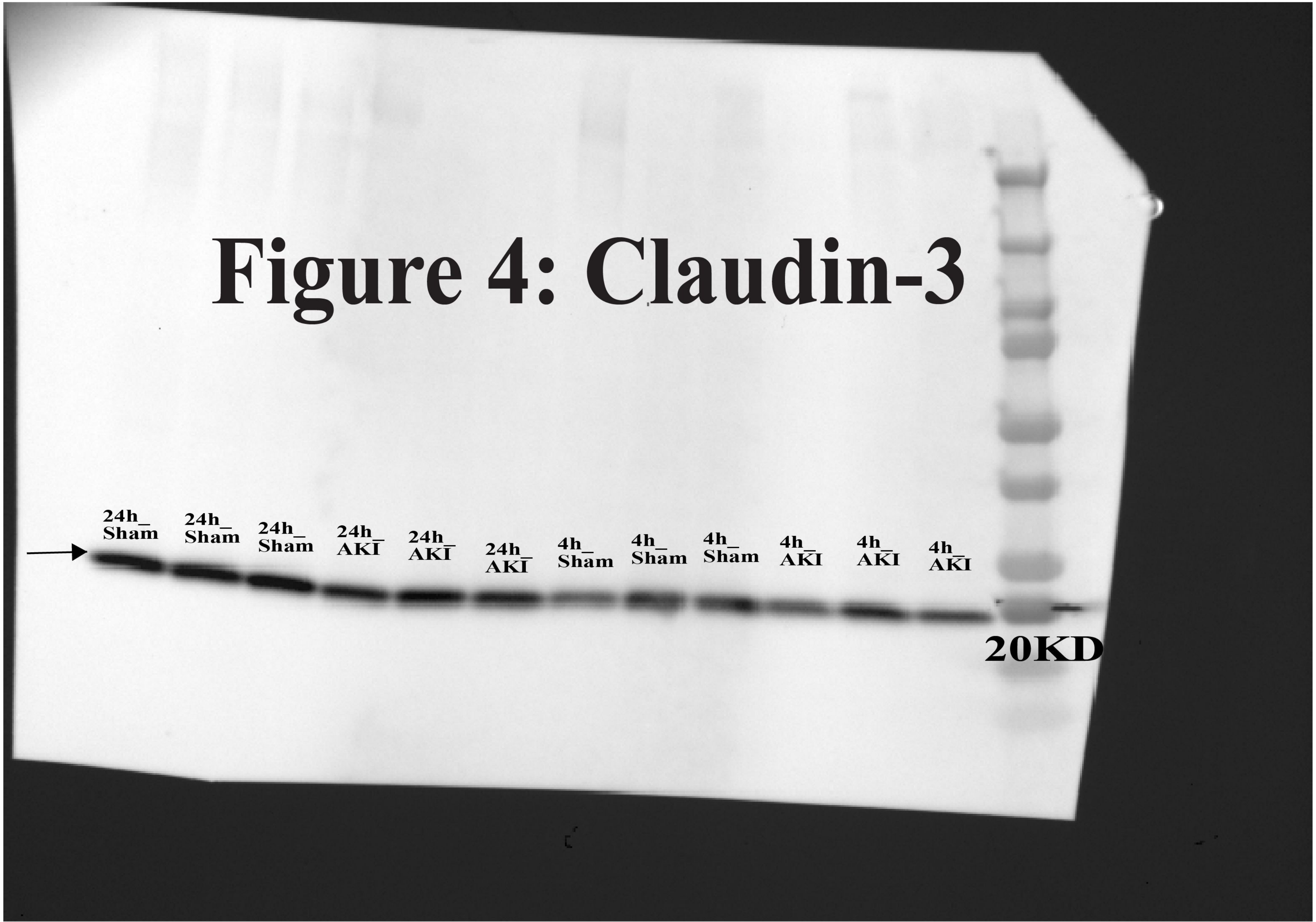

Figure 4: Claudin-4

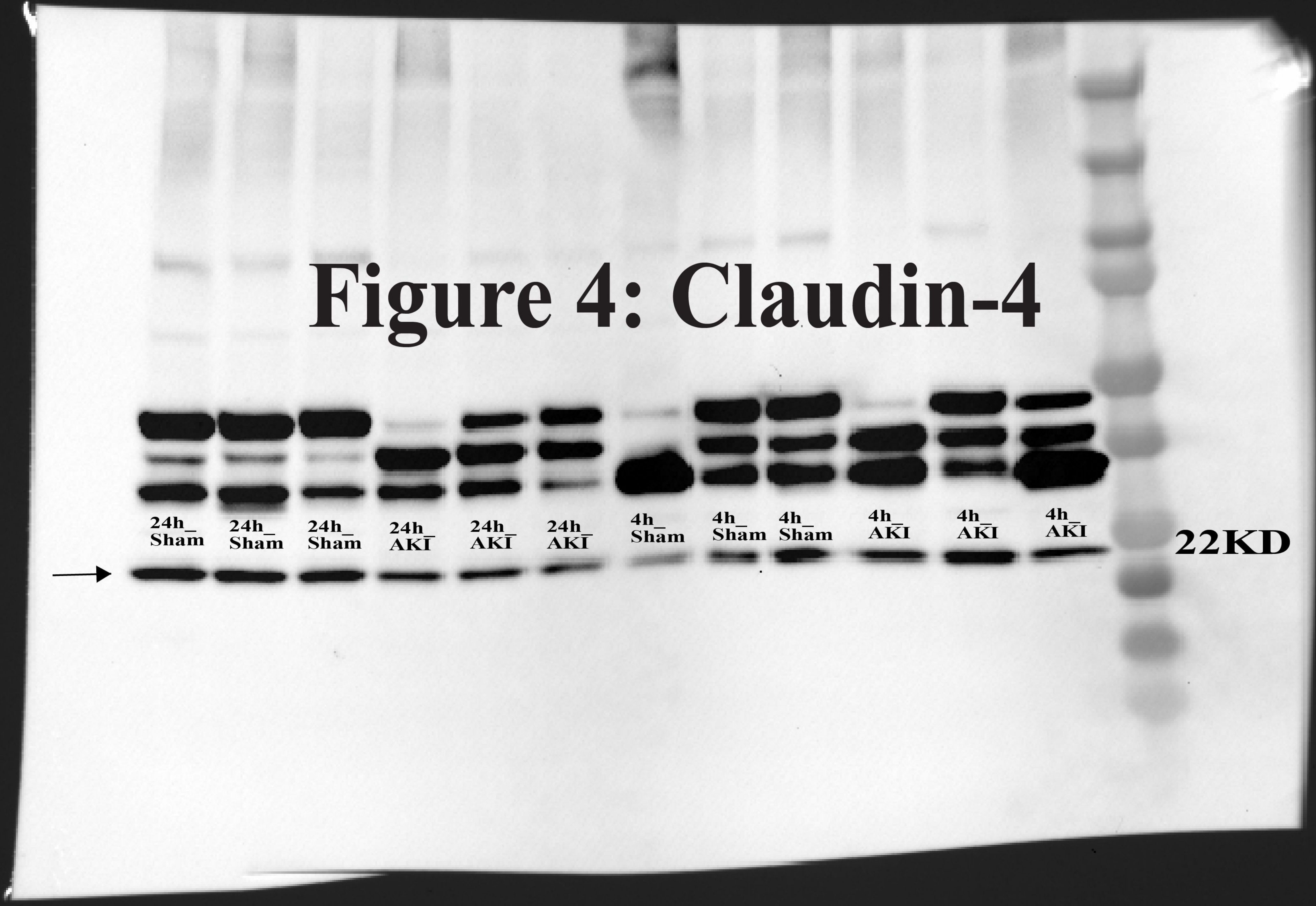

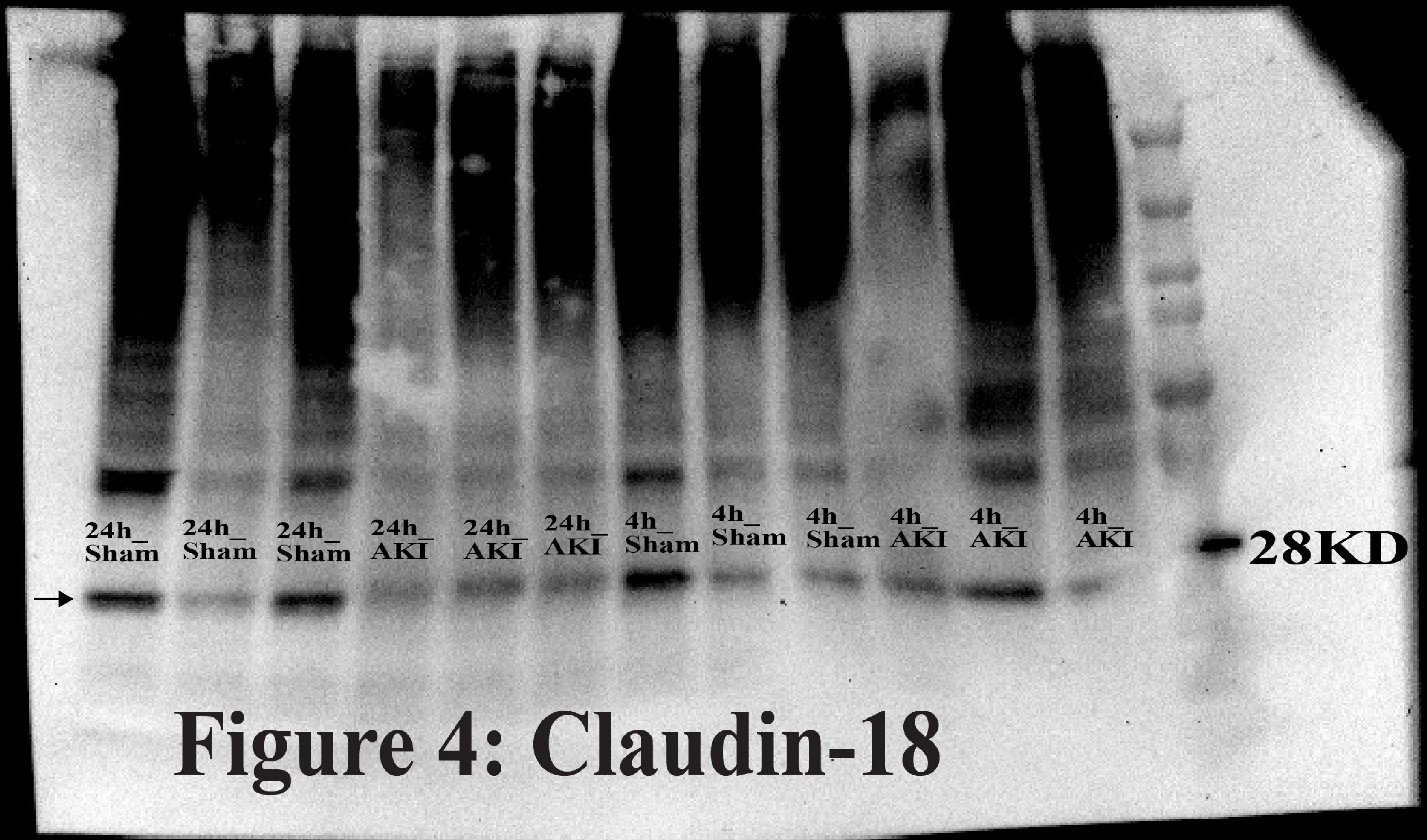

Figure 4: Claudin-18

Figure 4: JAMA-1

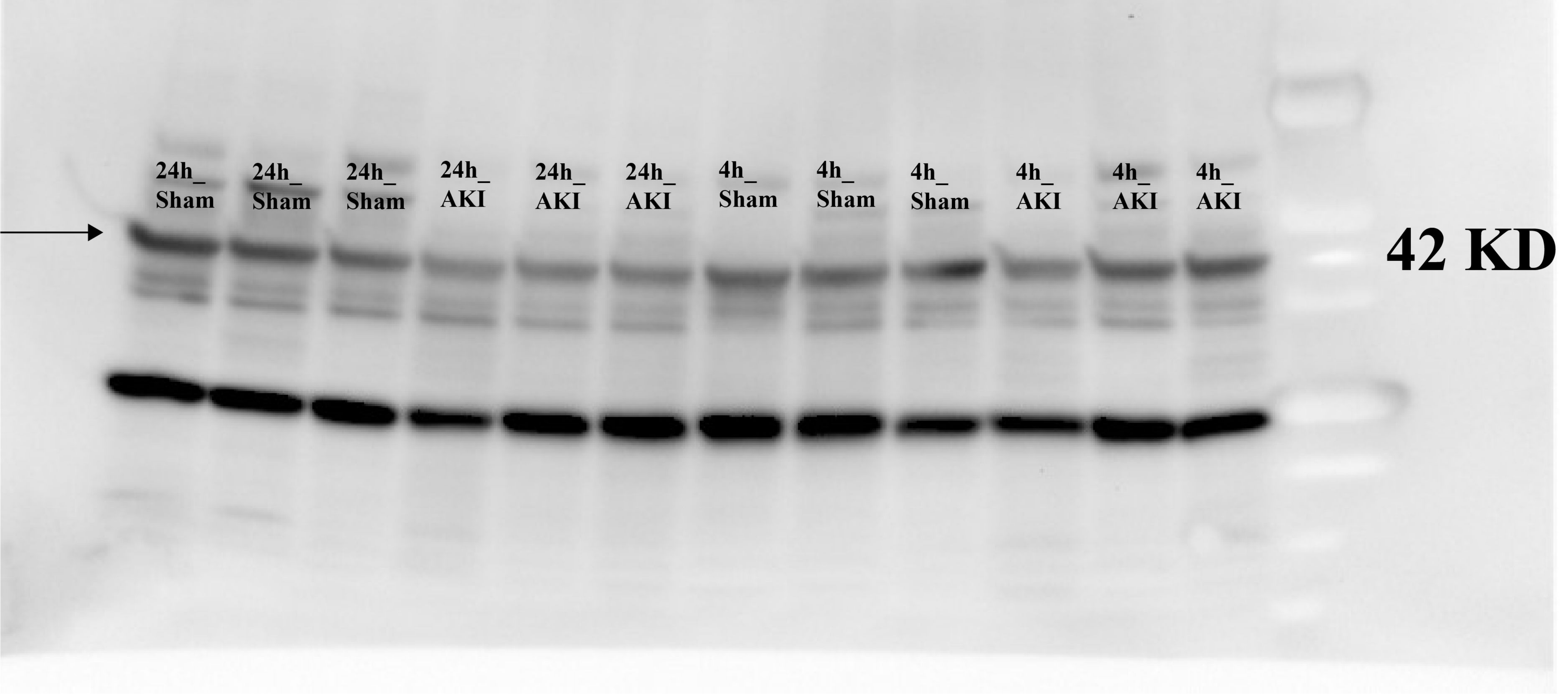

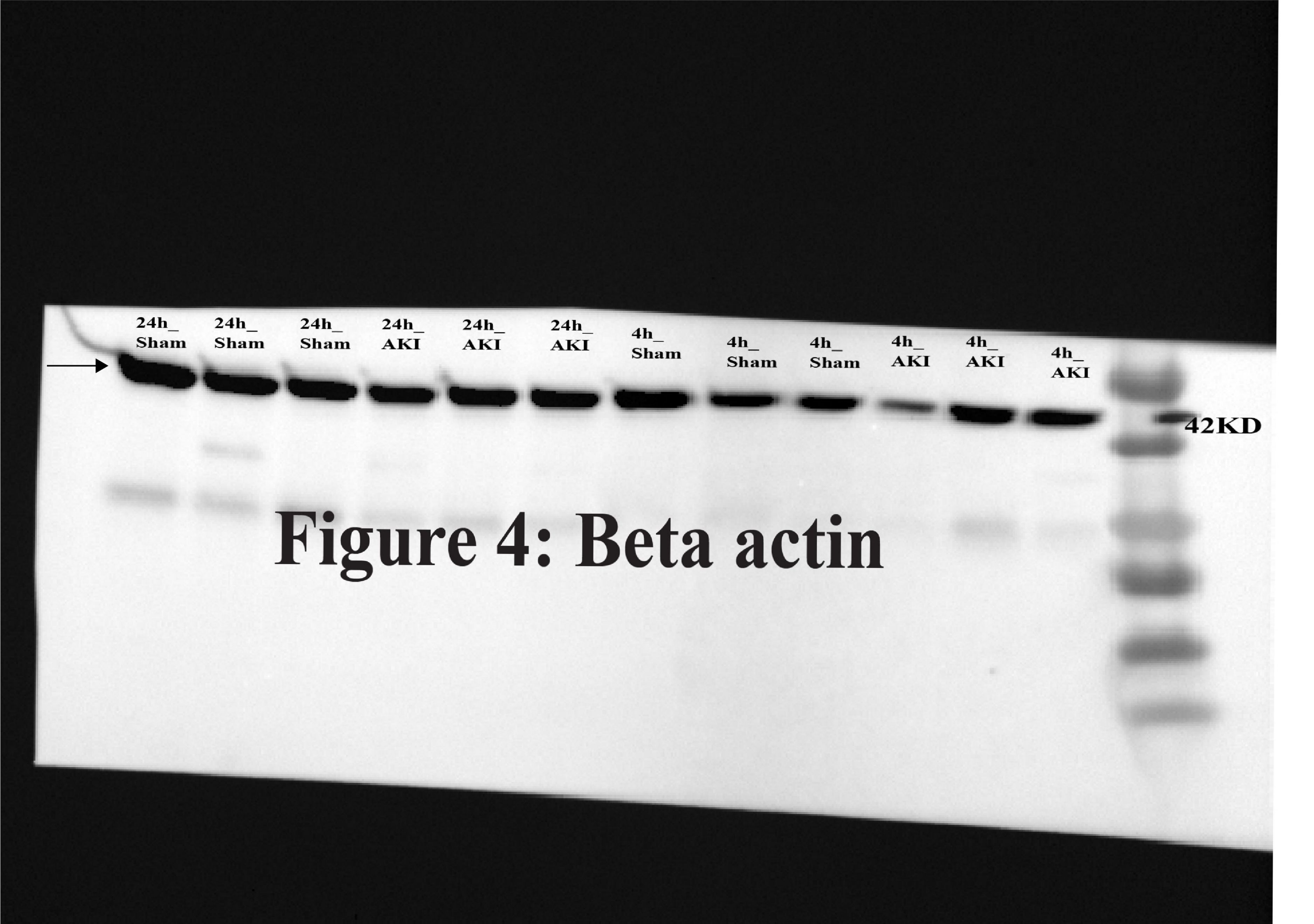

Figure 4: Beta actin
