# Supplementary material for: Change of surfactant protein D and A after renal ischemia reperfusion injury
Source: PLoS One. 2019 Dec 26;14(12):e0227097. doi: 10.1371/journal.pone.0227097 (PMC6932791; doi:10.1371/journal.pone.0227097)
Supplement: Attachment — Submitted filename: Response_to_Reviewers_20191115.docx [file pone.0227097.s005.docx]

Dear Partha Mukhopadhyay, Ph.D.

Academic Editor, PLOS ONE

Thank you so much for your comments. I am glad to see your comments, indicating that I have a chance to revise my manuscript. Following the reviewer’s comments, I have amended my manuscript as written below. In text, the changes and corrections have been highlighted by yellow color and green color changes mean deleted sentences. I hope you would review it again and I hope for the honor of accepting my scientific writing in your highly authorized journal.

**Reviewer 1 comments**

Reviewer #1: The manuscript entitled “Change of surfactant protein D and A after renal ischemia reperfusion injury” suggested a link between serum surfactant protein and renal ischemia reperfusion models. Furthermore, increased lung inflammation and apoptosis were observed due to change of lung tight-junction protein. The authors demonstrated the crosstalk between organs (kidney and lung), however, several concerns must be addressed.

Comment 1. The information in the manuscript does not provide a very well-explainable data between AKI and surfactant protein. Albeit further study will be required in the future, certain conclusion must be given in the manuscript. Therefore, it is pretty tricky to consider the overall ideas the authors provided. The authors need to provide schematic overview of these results for the readers to understand easily.

Answer: Thank you for your kind suggestion. A schematic overview has been added in the revised manuscript. Kindly, check the figure 7 in the revised manuscript.

Comment 2. There is too much information on materials and methods. The authors need to make it simplify.

Answer: We have simplified the materials and methods according to the suggestion in the revised manuscript. Some details of materials and methods have been transferred in supplementary document.

Comment 3. General figure image quality is too poor. It is very difficult to recognize clearly (histology, graph and western blot). It must be addressed to increase image quality (more than 300DPI).

Answer: We have improved the resolution of all figures. The image quality has been increased according to the suggestion in the revised manuscript.

Comment 4. In results part, the “sub-title” must summarize the results obtained. There is no information of the sub-title the authors provided in the manuscript. It will be re-organized.

Answer: The subtitles of the result part have been reorganized and the summarized result has been added to the result subtitles of the revised manuscript.

Comment 5. The methods used are described in FIGURE LEGEND. Do not state the results in the FIGURE LEGEND.

For example,

(FIG 1A) Renal function in C57BL/6 mice renal ischemia reperfusion blood urea nitrogen (BUN) and creatinine “increased” --> “evaluated”;

(FIG 1B) Tubular injury necrosis scores for PAS-stained kidney sections showed increased tubular injury in ischemic AKI --> kidney sections were subjected to PAS-staining and histological changes were scored.

Answer: The figure legends have been changed according to your comment.

Comment 6. Fig 3B and Fig4 Western blot images, the bands were cut group by group. The authors must provide the entire row immune-blotting bands. Do not cut (or split) the Western bands.

Answer: Thank you for the suggestion and the entire row immune-blotting bands has been provided according to the suggestion in the revised manuscript. When we did western blot, the band order is 24hr sham, 24hr AKI, 4hr Sham, and 4hr AKI. I am afraid that these band order make readers confused.

Comment 7. Figure1A, the order of graphs (BUN and creatine) should be changed. The manuscript stated serum creatinine and BUN (in the results). Try to make the consistency.

Answer: Thank you for the observation. The correction has been done in the revised manuscript.

Comment 8. Figure1B, please indicate the areas of tubular damage (i.e. using arrowhead)

Answer: Thank you for the suggestion. The arrow has been provided to indicate the areas of tubular damage in the figure 1B in the revised manuscript.

Comment 9. Line 182, “protein in BAL increased in the AKI”. Which protein? How to measure? Or any protein changes between shame and AKI models?

Answer: In previous manuscript “protein in BAL increased in the AKI” meant the “**total protein”** in BAL increased in AKI compared to sham. The total protein in the BAL of all sham and AKI models was measured by using Pierce BCA Protein Assay Kit (Thermo Fisher Scientific). The result showed that the amount of total protein in BAL was higher in AKI than sham.

Comment 10. There is no scale bars on Figures1, 3 and 5 (microscopic images)

Answer: The scale bars have been inserted.

Comment 11. In figure 2, put Blood serum on top of the graphs to recognize them easily.

Answer: The modification has been done in the revised manuscript according to the suggestion.

Comment 12. In figure 3A, how to measure neutrophil infiltration? By FACS or by count? If it was evaluated by count, only 1-2 neutrophils in the field? Make them more clarify.

Answer: Yes, we measured the neutrophil infiltration by counting and the count is correct. The pathologist scored the neutrophil count on a 400-fold slide as follows.

0 (rare neutrophils in capillary lumen), 1 (frequent neutrophils in capillary lumen), 2 (extravasation of neutrophils), 3 (aggregation of neutrophils in alveolar wall).

Comment 13. In figure 4 Western blotting, there is no information about the bands. Once again, do not split the western blot images.

Answer: Thank you for showing us the mistake and we believe it will help us to improve the quality of our article. The information and whole western blot picture have been added to the revised manuscript.

**Reviewer 2 comments**

Reviewer #2: The authors reported changes of SP-A and SP-D in an AKI mice model at different time points and compared serum and kidney SP-A & SP-D changes to other organ specific markers. They concluded that kidney-originated SP-A/D changes could not be ruled out and thus the two markers are unsuitable for determining AKI-related lung injury. The overall experimental design is adequate, however more details in animal experiments are welcomed. I have a few comments/questions:

Comment 1. In Materials and methods, please indicate sex of animals used.

Answer: In the revised manuscript we have added the sex (male) of the animals.

Comment 2. How was animal sacrifice performed? Spine dislocation or anesthetic overdose? Please specify.

Answer: We sacrificed our animals by spine dislocation method. In the revised manuscript the information is specified, kindly, find the added information in the revised manuscript.

Comment 3. Please define ‘80% return’ in BAL collection paragraph and specify pH or product number of PBS used.

Answer: Thank you for the question and it had helped us to solve the typing mistake. Previously we mistakenly wrote samples with 80% return were discarded instead of samples with **<80%** return was discarded. Normally, BAL contains different biochemical and cytological indicators that represent the status of whole lung but the BAL samples that are obtained from <80% return of lavage cannot exactly represent the whole lung status, so we discarded all the samples with **<80%** return of lavage.

In the revised manuscript we have corrected the information from 80% to <80% as well as specified the pH (7.4) and the product information (Gibco, USA) for the PBS.

Comment 4. Did you conduct PBS flush of lung vasculature before extracting BAL? why or why not?

Answer: We did not conduct PBS flush. We think that PBS flush could influence the tight junction barrier because of flush pressure. Also, we did follow previous reports (Kidney Int. 2017 Aug;92(2):365-376, Kidney Int. 2017 May;91(5):1057-1069.)

Comment 5. Which parts of centrifugated serum or BAL samples were kept? Please specify.

Answer: We stored the supernatant of the centrifuged serum and BAL. This information is specified in the revised manuscript.

Comment 6. In results:

In SP-A and SP-D assays, error bars are relatively outstanding. Specifically, Lung SP-A protein assay displayed inconsistency in 24h sham group, can the author address the cause of this anomaly? 4hr sham groups and 24h sham group underwent similar surgical procedures, and yet SP-A protein in kidney shows reduction in 24hr sham vs. 4hr sham. Which leads to suspicion that the ‘sham’ surgery is too invasive and disruptive to kidney function, Can the authors please address this phenomenon?

Answer: When we made the sham group, we tried to follow the same procedure as AKI model except for ischemic reperfusion. I have already published that Sham itself affect heart metabolites (Kidney Int. 2019 Mar;95(3):590-610). Although we had made effort to be less invasive when making sham, but sham operation itself could be engaged with injury. BUN, Creatinine and kidney histology are stable in both two shams.

Comment 7. In results:

In some data presentation, one group presents much more substantial error bar than other groups. In common knowledge, age and body weight-controlled mice are expected to have very consistent biological and biochemical profile under consistent modeling technique. I have to say that the data quality is questionable.

Answer: BUN/Cr showed similar values in same groups. But some surfactant protein values have wide range. This might be an individual difference in response to IR, but it is thought that this may be a wide range because the amount (pg/ml) of surfactant protein is very small compared to bun/cr (mg/dl).

Comment 8. In results:

In multiple graphs, SP-A and SP-D shows different trends, it would appear to me that SP-D responds in latent phase while SP-A has more timely response and increased very soon after ischemia reperfusion injury occurs. Why we should look at both targets at the same time, and how do we interpret the difference in their responses? I hope the authors can give a bit discussion in this aspect.

Answer: Thank you for your excellent comment. We checked surfactant protein A and D change after paraquat injection. (Korean J Intern Med. 2007 Jun;22(2):67-72.) The current pattern is similar with this study. I agree that SP-A has early response to stress ([Lab Invest.](https://www.ncbi.nlm.nih.gov/pubmed/8569184) 1996 Jan;74(1):209-20.). We have incorporated these in discussion section.

Comment 9. The topic brought up by the authors warrants attention, however the results are of less that satisfying quality. It would be great if well controlled experiments are performed and consistent data is presented, then the same conclusions will be much more convincing.

Answer: Thank you for the valuable comment. I think that further study needs more numbers of animals in each group and should uncover the role of surfactant protein in each specific organ. For the future direction, we will design experimental study to prove the crosstalk of surfactant proteins between kidney and lung in mice and validate this employing human data.
