# Supplementary material for: Change of surfactant protein D and A after renal ischemia reperfusion injury
Source: PLoS One. 2019 Dec 26;14(12):e0227097. doi: 10.1371/journal.pone.0227097 (PMC6932791; doi:10.1371/journal.pone.0227097)
Supplement: Attachment — Submitted filename: Response_to_Reviewers_20191210.docx [file pone.0227097.s006.docx]

Dear Partha Mukhopadhyay, Ph.D.

Academic Editor, PLOS ONE

Thank you so much for your comments. I am glad to see your comments, indicating that I have a chance to revise my manuscript. Following the reviewer’s comments, I have amended my manuscript as written below. In text, the changes and corrections have been highlighted by yellow color and green color changes mean deleted sentences. I hope you would review it again and I hope for the honor of accepting my scientific writing in your highly authorized journal.

**Reviewer 1 comments**

**Comment 1.**  In Fig 1A, Y axis of the graphs, mg/dL and mg/dl are mixed. Make them consistent.

**Answer:** Thank you for showing us the mistake and we hope it will help us to upgrade the quality of our manuscript. The correction has been completed and we have made **mg/dl** consistent in Fig 1A in the revised manuscript.

**Comment 2.**  In the manuscript, put the full name of ATN (might be “Acute tubular necrosis”), related with Fig 1B

**Answer:** Thank you for the suggestion. We have added the full name of **Acute tubular necrosis** (ATN) in the revised manuscript. Kindly check the revised manuscript, page 6, line 132 and 138.

**Comment 3.** In Fig 3A, H&E staining for neutrophil is not clear to determine NP infiltration. The evaluation of NP infiltration was done by pathologist according to the authors explanation and it was fully understandable. However, please perform MPO staining to confirm NP infiltration on those sites and put that data on Supporting figure?

**Answer:** Thank you for the good comment. I agree with your point. Unfortunately, there is a problem with the block, which is not suitable for MPO staining. Considering what we can technically do, we took the picture by **x 1000 times** and added as a supplementary figure in the revised manuscript. Kindly, check the supplementary Fig S1 in the supplementary document and additionally we have provided the picture below:

**Comment 4.** In Fig 4 (western blot), JAMA-1 or JAM-A? clarify

**Answer:** Thank you for showing us the mistake. In the Fig 4 it will be JAMA-1 (anti-junctional adhesion molecule 1) and we have corrected the Fig 4 in the revised manuscript.

**Comment 5.** In Fig 4, Claudin 3, Claudin 4, Claudin 18 and Claudin-3, Claudin-4, Claudin-18 are mixed. Make them consistent.

**Answer:** Thank you for the suggestion. In the Fig 4, the correction has been completed according to the reviewer’s comment.

**Comment 6.** In Fig 6, the staining SP-A and SP-D was not still clear. Please count SP-A and SP-D positive cells and add one other graph how many positive cells were counted in each group.

**Answer:** Thank you for the suggestion. We have counted the SP-A and SP-D positive cells per HPF. We also insert the additional graph in **Fig 6** in the revised manuscript.
